# Developing a Risk Reduction Support System for Health System in Iran: A Case Study in Blood Supply Chain Management

**DOI:** 10.3390/ijerph19042139

**Published:** 2022-02-14

**Authors:** Ali Sibevei, Adel Azar, Mostafa Zandieh, Seyed Mohammad Khalili, Maziar Yazdani

**Affiliations:** 1 Department of Industrial Management, Faculty of Management and Economics, Tarbiat Modares University, Tehran 14115-111, Iran; alisibevei@modares.ac.ir (A.S.); azara@modares.ac.ir (A.A.); 2 Department of Industrial Management and Information Technology, Management and Accounting Faculty, Shahid Beheshti University, Tehran 1983969411, Iran; m_zandieh@sbu.ac.ir; 3 Department of Industrial Engineering, Faculty of Engineering, Khayyam University, Mashhad 9189747178, Iran; m.khalili@khayyam.ac.ir; 4 School of Built Environment, University of New South Wales, Kensington, Sydney, NSW 2052, Australia

**Keywords:** supply chain risks, blood supply chain, interpretive structural modelling, social network analysis

## Abstract

Health systems are recognised as playing a potentially important role in many risk management strategies; however, there is strong evidence that health systems themselves have been the victims of unanticipated risks and have lost their functionality in providing reliable services. Existing risk identification and assessment tools in the health sector, particularly in the blood supply chain, address and evaluate risks without taking into account their interdependence and a holistic perspective. As a result, the aim of this paper is to develop a new systemic framework based on a semi-quantitative risk assessment approach to measure supply chain risks, which will be implemented through a case study on the Iranian BSC. This paper identifies and assesses supply chain risks (SCRs) by employing a novel systemic process known as SSM-SNA-ISM (SSI). First, the supply chain and its risks are identified using Soft Systems Methodology (SSM). Then, given the large number of risks, the second stage uses Social Network Analysis (SNA) to identify the relationships between the risks and select the most important ones. In the third stage, risk levelling is performed with a more in-depth analysis of the selected risks and the application of Interpretive Structural Modelling (ISM), and further analysis is performed using the Cross-Impact Matrix Multiplication Applied to Classification (MICMAC). The study found that by using the new proposed approach, taking into account risk relationships, and taking a holistic view, various supply chain risks could be assessed more effectively, especially when the number of risks is large. The findings also revealed that resolving the root risks of the blood supply chain frequently necessitates management skills. This paper contributes to the literature on supply chain risk management in two ways: First, a novel systemic approach to identifying and evaluating risks is proposed. This process offers a fresh perspective on supply chain risk modelling by utilising systems thinking tools. Second, by identifying Iranian BSC risks and identifying special risks.

## 1. Introduction

While the supply chain (SC) concept has attracted interest among academics, policymakers, and companies [1,2,3], its success in any organisation may be compromised by a variety of factors, including high demand volatility, short product life cycles, and varying customer requirements [4,5]. Addressing these issues is difficult due to the complex nature of SCs [6]. Hendricks and Singhal [7] asserted that due to a lack of proper risk management, SC issues may significantly reduce profits. In general, risk management entails identifying, analysing, and controlling risks [8]. Identifying and mitigating risks in SCs is accomplished not only at the company level, but also by focusing on the entire SC [9]. SCs are vulnerable to a variety of sources of uncertainty [10,11]. With the rise in uncertainties and risk events, supply chain risk management (SCRM) has received more attention than ever before and has become critical to business success [12,13]. Therefore, organisations need to learn how to mitigate the adverse impacts of risks associated with supply chain [14].

SCRM is being explored in many sectors such as health systems; however, some of them are more vulnerable due to the high complex interactions between their components [15,16,17]. Furthermore, unpredictability makes it difficult to manage and meet the needs of the patients. As a result, the unpredictability of SCs creates risks that have a direct impact on people’s lives. The performance of blood-related services in healthcare systems, on the other hand, is critical [18]. Because blood is the fluid in the body that supplies the necessary substances to the human body, it plays an important role in experiences such as accidents or emergencies [19]. Human disease is causing an uncontrollable demand for blood. It also comes from donors who volunteer to donate, so variations in blood supply and demand are unavoidable. Because blood has a short life span, it is a perishable product, and the blood centre should manage and distribute blood products in a complex, uncertain environment, which may pose a risk to the blood SC (BSC). There are many unanswered questions about the blood supply chain that need to be addressed, particularly in terms of risk management.

While numerous researchers have recognised the important role of reliable blood supply chain systems in the health sector, little research has been conducted on these systems’ vulnerability to providing reliable services. Blood supply chains have always been associated with a wide variety of risks, particularly in countries with poorly planned health systems, which should be identified and addressed concurrently. While the majority of studies have focused exclusively on one of these factors, more holistic approaches are required. Numerous techniques have been used, including the Delphi method, checklists, module decomposition, and scenario analysis. However, because there is no one-size-fits-all approach to risk identification, finding novel approaches is always an open research avenue. Additionally, many studies have used tools such as Failure Mode and Effects Analysis (FMEA) and multi-criteria decision-making (MCDM) during the risk assessment stage, but these methods do not take into account the relationship between risks. Additionally, when the number of identified risks is limited, methods such as the Analytic Network Process (ANP) or Interpretive Structural Modelling (ISM) are used, which make these methods unsuitable for the blood supply chain.

By addressing the aforementioned gaps, this study aims to add new theoretical and practical insights to a growing body of knowledge about reducing risks associated with blood supply chains in health systems, particularly in countries where the health sector is becoming increasingly vulnerable to unexpected risks. Therefore, the main contributions can be summarised in the following way.

This study contributes to the growing body of knowledge by proposing an integrated approach based on SSM, SNA, and ISM methods to identify and evaluate risks associated with the blood supply chain and then implement the proposed approach in a real-world case study. Risks are identified in the proposed system by creating a rich image that is then used to answer the preceding questions within SSM. Rich images offer a comprehensive and macro view of the situation. Thus, the blood supply chain and its components are depicted, followed by identifying the risks associated with the segments. The following step examines the relationships between identified risks in order to analyse and evaluate them. Since SNA is capable of analysing multiple variables, the most significant risks are identified using a relationship-based approach. The ISM method is then used to determine the relationships between the reduced key risks, allowing for a more in-depth analysis.

Due to the importance of SCRs particularly in healthcare SCs and the problem of risk assessment techniques when the number of risks is high, “The central aim of this study is to develop an approach to support decision makers in coping with the challenge of risks currently faced by health systems to evaluate risks by taking a holistic view of the BSC using systemic tools”. The research questions that are addressed by this study are:

*Question 1:* How can we identify risks in a systemic way?
*Question 2:* How can we evaluate the high number of risks regarding their relationships?
*Question 3:* What are the most important SCRs in Iran’s blood SC?

The paper has been structured as follows: Section 2 provides a literature review about blood SCRs and risk assessment techniques. Section 3 deals with methodology and provides information about each step. Section 4 presents the practical implementation of the new proposed methodology in the Iranian BSC. Finally, the conclusion and managerial insights are explained in the last section.

## 2. Literature Review

Below is a review of the related literature in two distinct but related research streams.

### 2.1. Blood Supply Chain

#### Healthcare and Blood Supply Chain

Healthcare systems are becoming increasingly complex and uncertain. Every step of the healthcare decision-making process involves an element of uncertainty. There are numerous risks associated with all aspects of healthcare, from diagnosis to treatment to rehabilitation. Incorrect assessments can have unintended negative consequences that, by the time they are discovered, have already had an impact [20]. Healthcare risks are characterised by their complexity, the possibility of patient harm and uncertainty, and the possibility of serious outcomes with serious consequences. According to the US Institute of Medicine, approximately 10% of patients admitted to hospitals experience adverse events, the majority of which are preventable [21]. Blood and its derivatives are among the most critical items used in healthcare because there is no substitute [19]. Any patient’s treatment can be harmed by a lack of blood. The expiration of blood will result in an increase in the cost of blood transfusions [22]. There are several potential risks associated with blood function that can impair the healthcare network’s overall performance [23].

### 2.2. Risk Assessment Techniques

This section reviews important studies on SCRs in healthcare and other fields. In the first part, a literature review on healthcare and blood supply chain risks is presented and in the second part other areas are reviewed.

#### 2.2.1. Healthcare and Blood Supply Chain

Lu et al. [24], in an article using PDCA and FMEA methods, studied the RPNs before and after recovery while discussing the failure states of the blood transfusion process in China. Dehnavieh et al. [25] conducted a study to actively assess the risks of a blood transfusion process in a paediatric emergency education centre using the Health FMEA (HFMEA) method. Najafpour et al. [26] evaluated the blood transfusion process in a public hospital using FMEA. The results showed that mistransfusion is the most critical risk leading to severe morbidity or mortality. Cagliano et al. [27] merged three well-known risk management tools, namely Risk Breakdown Structure (RBS), Risk Breakdown Matrix (RBM), and FMECA, to provide a structured approach for identifying and analysing risk-related causes. Achmadi and Mansur [28] attempted to minimize the level of effective risk in the BSC using House of Risk (HOR). The results showed that eleven risk factors were needed to be prioritized by seven possible mitigation measures. Jaberidoost et al. [29] assessed risks in the pharmaceutical industry in Iran concerning the process, priorities, hazards, and risk probability. Risk analysis was performed using Analytic Hierarchy Process (AHP), rating scale, and SAW. Boonyanusith and Jittamai [23] examined the risks involved in the BSC to assess risks and evaluate their management practices using HOR. Mora et al. [30] used FMEA to evaluate potential failures and improve blood transfusion safety in an urban hospital. Each failure condition was evaluated using the probability of occurrence, severity, and probability of detection. Liu [20] proposed a new risk priority approach combining cluster analysis and prospect theory with FMEA when a large pool of experts was present. They also proposed an entropy-based approach to objectively weighing the risk factors using risk assessment information.

#### 2.2.2. Other Areas

Risk assessment and identification studies are presented in other areas in the following. Gaudenzi and Borghesi [31] used the AHP method to identify SCR factors to improve customer value. Wu et al. [32] used AHP to rank suppliers’ risk factors and develop a method for hierarchically classifying inbound supply risk factors. Pujawan and Geraldin [33] developed a framework for actively managing SCRs using HOR and presented a combination of core ideas from two well-known house of quality tools and FMEA. Moeinzadeh and Hajfathaliha [34] employed a combination of fuzzy sets, ANP and VIKOR for SCR assessment. Wang et al. [35] presented a risk assessment model capable of the structural analysis of aggregative risk implementation of various green schemes in the fashion SC. They used fuzzy AHP to calculate Aggregative Risk Index (ARI). Song et al. [36] investigated the major risk factors of Sustainable SCM (SSCM) based on rough logic and Decision-Making Trial and Evaluation Laboratory (DEMATEL) method. Curkovic et al. [37] tried to find out how corporate SCRs are managed with a particular focus on the use of FMEA and how FMEA can play an important role in the process of risk management through supplier evaluation and selection. Nazam et al. [38] used a fuzzy AHP to calculate the weight of each of the risk criteria and proposed the TOPSIS technique for ranking and evaluating the related risks in a green SC. Fazli et al. [39] identified the major risks associated with crude oil SC. They used DEMATEL to determine the interdependence between risks and then used ANP to assess the significance of each risk. Rostamzadeh et al. [40] developed a framework for sustainable SCRM (SSCRM) using TOPSIS and CRITIC. Taking a holistic view, Junaid et al. [41] combined neutrosophy with AHP and TOPSIS and implemented this hybrid approach in an automobile industry. A summary of the previous studies presented in the Table 1.

A review of the blood-related literature indicates that most of the previous studies focused on a part of an SC and only a few studies have discussed the issue of risk in the blood SC [23,28,29]. Furthermore, only a few studies in this field, such as Jaberidoost, Olfat, Hosseini, Kebriaeezadeh, Abdollahi, Alaeddini, and Dinarvand [29], have used MCDM methods to identify risks. To study the methods used to identify and evaluate risks, SCRs have been studied in other areas as well. The results show that most of the studies have employed MCDM methods such as CRITIC and TOPSIS [40], ANP and VIKOR [34], and AHP TOPSIS [38]. Whereas in our proposed hybrid approach, the SC is first investigated using SSM and the risks are identified. Then, the combined SNA-ISM approach is used due to the high number of risks and taking the relationships among them into account. Many of the approaches mentioned in the above research works are not capable of considering relationships or are not suitable for a high number of risks; the novelty of the presented work is to achieve these two issues utilizing the SNA-ISM hybrid approach. According to our findings, no study has used the proposed method yet, and it can be said that the present study is innovative from this perspective.

## 3. Methodology

**Figure 1:**
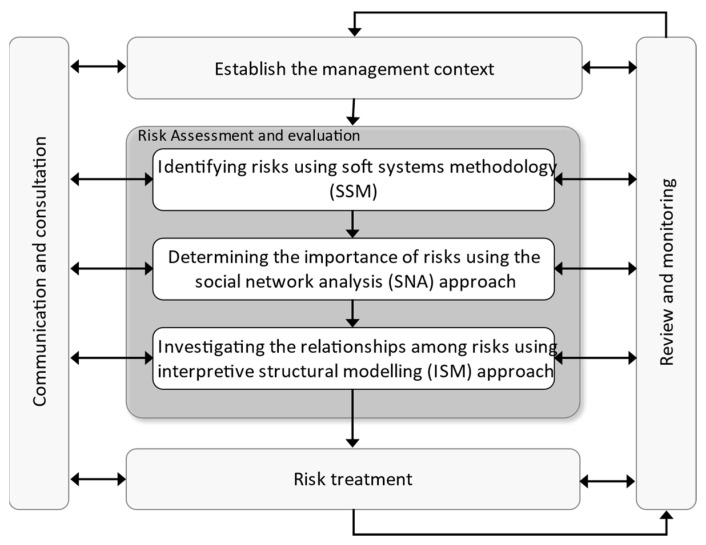
Steps of the proposed framework.

This study presents an integrated framework based on qualitative and quantitative approaches to identify and assess risks in the BSC. Contextualization entails defining the risk management process’ scope, defining the organization’s objectives, and establishing risk evaluation criteria. The context is made up of external factors (regulatory environment, market conditions, stakeholder expectations) and internal factors (governance, culture, standards and rules, capabilities, existing contracts, employee expectations, information systems, and so on). In the first step, the qualitative analysis uses the rich picture within SSM to identify the risks of the BSC. Thus, according to the internal and external categorization [43], the internal and external segments’ relations in the BSC are identified using the rich picture. Then, identifying the risks is done by reviewing the literature and interviewing the experts. Purposive sampling was used to select experts for this study. The experts included the general manager of Iranian blood transfusion, the manager of the IT department, 2 people from the blood donation department, the equipment technical managers (2 people), 2 people from the quality assurance department, 2 people from the blood bank department, and the accreditation manager of the blood transfusion organization. As mentioned earlier, when the number of risks is high, MCDM methods are not efficient, so in the second step of quantitative analysis, using the SNA approach, the importance of risks is derived based on their relationships by the degree of centrality index. Then, using the quartile score, the highest degrees are determined. If the degree of centrality is higher, the risk becomes more important, so the most important risks in the SC are those that are in the last quartile (high-risk) and are selected for further analysis. In the third step, the ISM approach examines the relationships among these key risks. The research steps that describe the methods are illustrated in Figure 1.

Monitoring and reviewing include assessing risk management performance with indicators that are assessed on a regular basis for adequacy. It entails checking for deviations from the risk management program, determining whether the risk management process, strategy, and plan are still suitable in light of the organization’s external and internal perspective, reporting on risk, progress with the risk management plan, and how well the risk management policy is being followed, and reviewing the risk management framework’s effectiveness. Communication and consultation aid in understanding stakeholders’ interests and concerns, ensuring that the risk management process is focused on the relevant parts, and explaining the reasoning for choices and specific risk treatment alternatives.

### 3.1. Identifying Risks Using Soft Systems Methodology (SSM)

**Figure 2:**
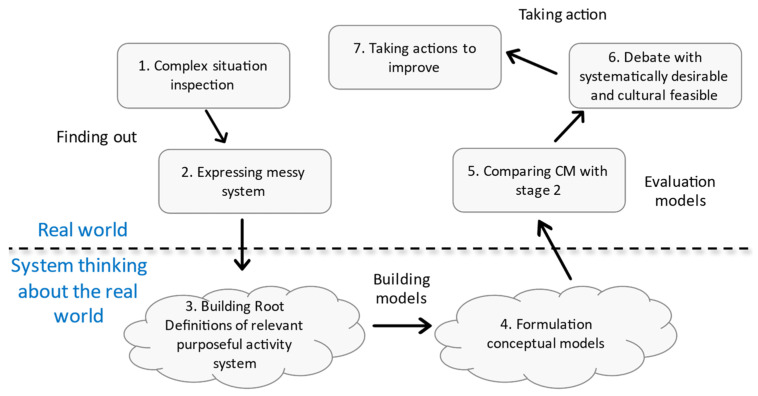
Seven steps of SSM adopted from Wang, Liu and Mingers [45].

Soft systems methodology (SSM) is a widely used methodology to analyse and solve problems in complex conditions [44]. As shown in Figure 2, this methodology consists of seven stages and organizes the way we think about complex situations so that action can be taken to improve them [45]. SSM uses “systems thinking” in a cycle of action research, learning, and reflection to help understand the various perceptions that may exist in the minds of different people involved in the situation [46]. Creating a rich picture is one of four problematic situation recognition techniques that have emerged from many successful tests and have become a common component in the SSM method [44]. Drawing rich pictures is a good way to show the relationships aiming to make sense of the system and to access key elements, structures, and perspectives in a situation [47]. The picture is much more useful than the written explanation, as it leads into a discussion on a level far greater than usual [44]. As mentioned, few studies have been conducted on the blood SC. Therefore, checking for a complete understanding of the components and connections of the BSC can be helpful. To identify the risks of a blood SC, a rich picture of this chain can provide better insights for experts. Hence, in this study, using the rich picture within the SSM approach, different sections related to the BSC are determined to identify their risks. Then the risks in each of the segments are identified using interview sessions and a research literature review.

### 3.2. Determining the Importance of Risks Using the Social Network Analysis (SNA) Approach

At this stage, we attempt to identify the most important risks using the degree of centrality analysis in the SNA approach. Degree of centrality: the simplest definition of centrality is that the central node is the most active one and has the most connections to other nodes [48]. The degree of a node is used to describe the total relations of that node with other nodes, or the number of edges adjacent to that node [49]. In the present study, from a conceptual point of view, having a higher degree of centrality indicates a risk that is most closely linked to other risks.

In this approach, the network is usually represented as a graph consisting of links by lines and nodes by points [50]. For centrality analysis, nodes are important; they are influenced by the degree of centrality. The centrality index is a method of measuring the number of links to a node [51]. In fact, the centrality of a node indicates the importance of that node as a whole [52]. Centrality is one of the most widely used indicators in SNA and represents what entity in the network is the core or has the most power [53].

Method of collecting data in SNA: the relational data required in this research were obtained by asking the experts about the relationship of each segment’s risks to each other and their relationship to the risks of other segments in a focus group meeting. In total, 11 experts were selected among the experienced managers in the Iranian BSC.

### 3.3. Investigating the Relationships among Risks Using ISM

The Interpretive Structural Modelling (ISM) methodology was first proposed to deal with complex problems. ISM is an analytical method that allows us to develop a structure of all existing relationships among the various components of a complex system [54]. The primary goal of such a model is to use experts’ knowledge and expertise to analyse complex system issues and then create a multi-level structural model [55]. The ISM approach is to be used by following several well-defined procedures in the sequence listed below. Each step is important and connected to the one before it, and it cannot be skipped [56]:

1. *Identification of criteria:* The elements are selected based on their relevance to the problem, so the first step is to identify them. In this step, the key risks identified in the previous step are used.
2. *Establishing the relation between dimensions and indicators:* It expresses the relationship between variables. Such relationships can have a wide range of consequences (influencing, comparative, temporal, or neutral).
3. *Construction of the Structural Self-Interaction Matrix (SSIM) by pairwise comparison:* The participants should decide on the pair relationship between the variables during this step. The relationship between two variables i and j is investigated, as well as the direction of the relationship. The four symbols used to indicate the direction of the i and j relationship are as follows:
  - V: variable i leads to variable j.
  - A: variable j leads to the variable i.
  - X: a bidirectional relationship (from i to j and from j to i)
  - O: no relationship between the variables.
4. *Development of a reachability matrix from SSIM and transitivity check:* This step is related to building a reachability matrix. Since this is a binary matrix, the inputs V, A, X, and O of SSIM are converted to 1 and 0.
5. *Level partition on reachability matrix*: The reachability matrix is classified at different levels.
6. *Development of the digraph*: Elements are graphically arranged in levels and links are drawn according to the relationships shown in the reachability matrix.
7. *Interaction matrix*: The final digraph is transformed into a binary interaction matrix that represents all relationships with input 1.
8. *Diagraph formation and its conversion*: The digraph is converted to ISM and examined for conceptual inconsistency.
9. *MICMAC analysis*: The purpose of this analysis is to identify and analyse the driving and dependence power of variables. So, the variables are divided into four categories of autonomous, dependent, linkage and independent drivers in terms of the driving and dependence power.

### 3.4. Strategies for Threats

Various strategies have been proposed in the literature for risk treatment and response, most of which have emphasized risk mitigation [57,58]. Here are five common types of treatments [59]:

- Risk acceptance: This strategy means that because of, for example, high costs, risk is accepted, and nothing is done about it [60].
- Risk avoidance: This strategy seeks to eliminate the types of events and root causes that trigger risk [61].
- Risk transfer: This strategy means delegating responsibility to another group. They are especially suitable for disruption risks such as natural disasters, which have a low probability and high impact (Zhen et al., 2016).
- IRisk sharing: This strategy means sharing some or all of the risks with another party, which is usually done through contracting by other companies [62].
- Risk mitigation: This strategy seeks to reduce risks to an acceptable level [63].

## 4. Practical Implementation

The research methodology states that this study uses SSM, SNA, and ISM to identify the relevance of and correlations between risks. As shown in Figure 1, this section is broken down into three sections. The main aim of this section is to assess the proposed model using a case study. Therefore, some health centres, which are highly exposed to risks, are analysed. In addition, a comprehensive sensitivity analysis of some factors is conducted to provide insights into how the performance of the system can be enhanced.

### 4.1. Identifying the Risks in the BSC Using SSM

**Figure 3:**
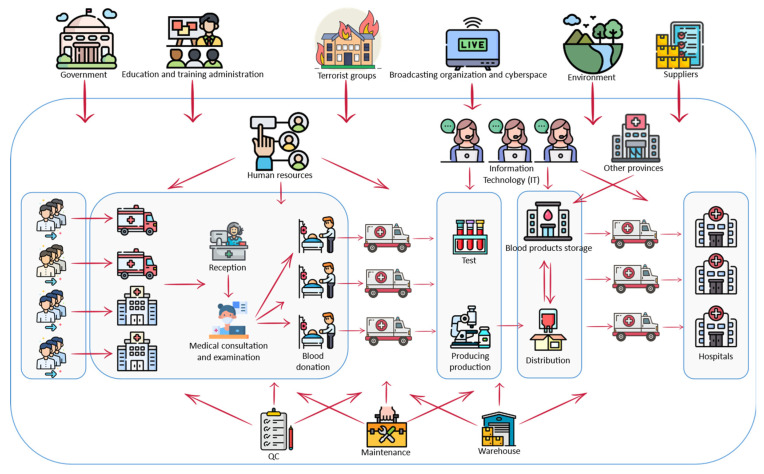
The rich picture of the BSC.

SSM is first used to identify the SC and the set of relationships it has with other departments, as shown in Figure 1. In fact, a detailed picture is used to aid in risk identification. So, once the sections related to the blood SC are determined, the risks in each of these sections are determined. For example, the relationship between the government and the BSC is determined using the rich picture in Figure 3. Following that, the risks associated with each sector are identified.

The Blood SCRs are categorized according to the identified segments of SSM. As shown in Figure 3, firstly, the relevant sections in the BSC have been drawn to recognize the SC completely, then the risks of each segment have been identified using expert opinions. Table 2 indicates the identified segments for a total of 102 risks.

### 4.2. Identifying the Important Risks by Using SNA

**Figure 4:**
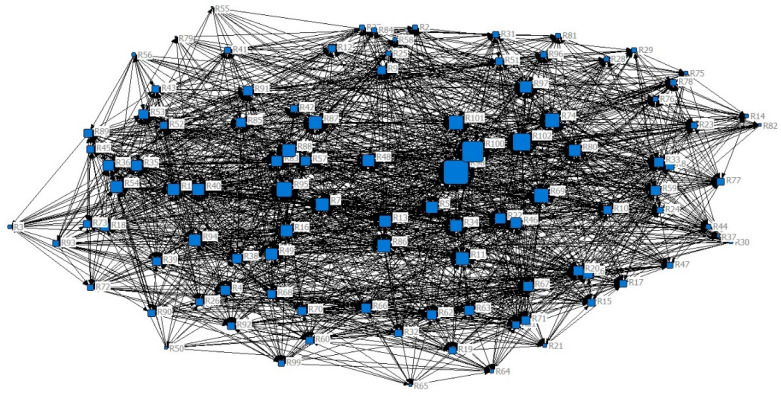
Relation among risks according to the degree of centrality.

The importance of the previously identified risks will be determined using the SNA method at this stage. UCINET and NetDraw software were used for this purpose. Experts determined whether or not any identified risk was related to each other in order to collect relational data. As a result, the risk communication matrix was entered into the UCINET software as an input, and the relationship network was plotted in the NetDraw software. The two software facilities were then used to calculate and analyse various values of centrality in the network. Figure 4 depicts a graph of NetDraw software’s degree of centrality. The larger the size of each node, the greater the degree of centrality of that node.

The degree of centrality in the network of blood SCRs indicates how each risk is related to other risks. The higher the centrality index, the more closely related that risk is to other risks. The risks were then sorted according to their centrality index using the Q score. The risks in the upper quartile are the most significant and have the highest degree of centrality. In other words, because of their relevance to other risks, these risks are considered key risks in the network and are thus chosen for further analysis and evaluation. For a better understanding of Figure 4, Table 3 is presented to show the degree of centrality of all risks.

As shown in Figure 4 and Table 3, the risk of low employee productivity has the highest degree of centrality; that means this risk can affect or be affected by many other risks.

### 4.3. Determining the Relationship among Risks

On the most important risks identified, a more detailed analysis of relationships and classification is required. These risks were incorporated into the structural self-interaction matrix. To accomplish this, a questionnaire with 17 risks was produced, and experts were asked to identify the relationships between the risks. The experts completed the structural self-interaction matrix by utilising the four conceptual relationship modes (Table 4).

By converting the SSIM into a binary matrix, the initial reachability matrix was obtained. Following the formation of the initial reachability matrix, the final reachability matrix was created in the same manner as shown in Table 5.

**Figure 5:**
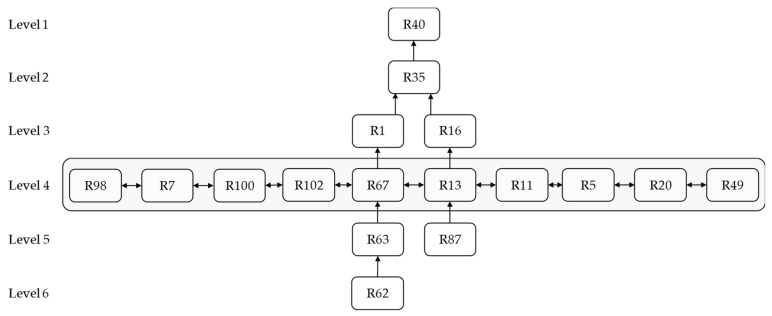
ISM-based model of BSC risks.

The final reachability matrix must be categorized into different levels. Therefore, in this study, according to the levels of risks and the final reachability matrix, the final model was drawn, which consisted of six levels. It is to be noted that, as shown in Figure 5, higher-level risks are less effective and more likely to be affected by other risks. For example, insufficient response to the hospital demand (R40) is at its highest level, so lower risk levels are more likely to be affected. In addition, sanctions’ economic and political effects are at the lowest level, indicating that they can affect other risks; hence, some approaches must be considered to address it.

### 4.4. MICMAC Analysis

**Figure 6:**
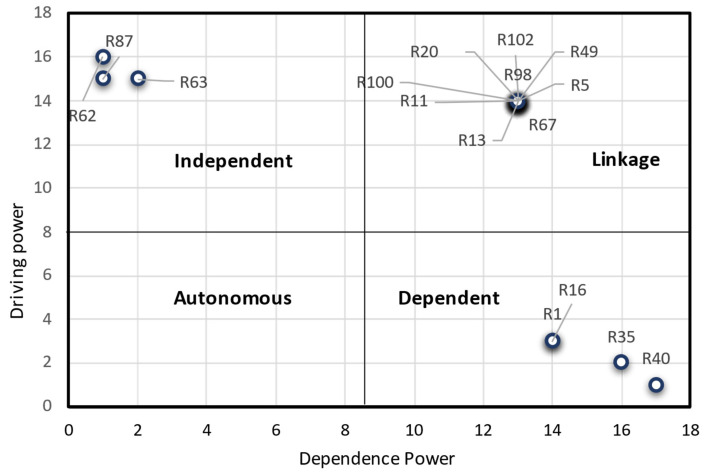
MICMAC analysis.

At this stage, using the MICMAC diagram and after determining the driving and dependence power of the risks, all key risks in the BSC can be organized into one of the four categories. The first group includes autonomous risks that are somewhat separate from other risks and have little relevance; there was no autonomous risk in this study. The second group belongs to dependent risks that have weak influence but high dependency. According to Figure 6, the dependent risks include an insufficient estimate of the amount needed to collect (R1), late delivery (R16), improper blood inventory level (R35), and insufficient response to hospital demand (R40) as the most affected by other risks. In other words, there are many factors involved in creating these risks, which are less likely to pose other risks. The third group consists of linkage risks with high driving power and high dependency. This group includes the risks of software problems (R5), equipment failure (R11), wastes and losses (R13), failure to perform preventive maintenance (R20), lack of materials and equipment (such as kits and bags) (R49), not paying attention to standards and validations (R100), reduction in employee productivity (R98), and selection of inappropriate suppliers (R67). The last group includes independent risks such as economic and political effects of sanctions (R62), changes in the exchange rate (R63) and earthquakes (R87). These risks have high driving power and low dependency. Risks with high driving power are called key risks, which fall into one of the two groups of independent risks or linkage risks. In fact, any changes to these risks can change other risks.

## 5. Discussion and Conclusions

### 5.1. Discussion

BSCs are exposed to a variety of risks in many health systems, and their neglect can result in a slew of complications. SCRM, which has grown in popularity over the last few years, can assist managers in better preparing for future events by identifying and analysing risks within the SC. Due to the importance of this research area, this research proposed a new systemic approach (SSI) for identifying and analysing SCRs. By first taking a holistic view and conducting a more comprehensive study of the BSC and its components, as well as by utilising a rich picture within SSM, risks associated with each segment were identified throughout the SC with the assistance of experts and previous articles. Then, as previously stated, due to the critical nature of relationships in evaluating SCRs and the large number of identified risks, SNA was used to ascertain the relationships among the identified risks and to select the most significant ones. Finally, the ISM approach was used to examine the levelling and establish bilateral relationships between the key risks.

By drawing the rich picture of the Iranian blood SC, the whole blood SCRs could be better identified, and 102 risks were extracted. An analysis of risk relationships was conducted utilising SNA and the input of industry experts, and then the most important risks were chosen. Risks include low productivity of employees, earthquakes, changes in the exchange rates, an insufficient response to hospital demand, and late delivery of the blood bags, to mention but a few. In fact, given the high relation of these risks, managers should be particularly concerned with identifying appropriate strategies for mitigating them. Then, ISM and MICMAC analyses were conducted for the key selected risks. Risks such as the economic and political effects of sanctions, changes in the exchange rate and earthquakes have high driving power and low dependency. Management should consider these risks because of their high impact on other risks, as determined by the MICMAC analysis report. Due to the lack of management control over these risks, managers need to enact policies specific to dealing with them. Particularly, an earthquake is a disruption event and is unpredictable, so special strategies such as flexibility and agility in the transportation systems and redundancy in inventory and suppliers are required to make the SC resilient. Another important point regarding the management of risks in health systems is that these risks are not medical, so the top managers of the BSC must also take general management courses, particularly risk management. Higher levels of risk include, for example, late delivery, incorrect blood inventory levels and inadequate estimations of the amount needed to collect blood. Blood-related risks, which can be influenced by other risks, appear in the dependent group. Because of their importance and direct effects on human life, they must be regarded in particular.

Risks such as earthquakes (R 87), exchange rate fluctuations (R 63), and the political consequences of sanctions (R 62) are among the risks identified as independent variables in the MICMAC matrix. There are two critical points to consider regarding these risks. To begin, these are the most effective and least dependent risks. Second, these risks are out of the organization’s control and are frequently referred to as disruption risks. Risk sharing or risk transfer may be the appropriate strategy for the organisation to pursue. Additionally, without a doubt, managers should consider making supply chains resilient, given the critical nature of resilience in today’s world. Naturally, in the event of an earthquake, numerous articles have been reviewed to strengthen the blood supply chain’s resilience. For the other two risks, namely the political consequences of sanctions and the exchange rate, focusing on domestic production may be an advisable policy. On the other hand, risks such as late delivery (R16), insufficient blood inventory (R35), and insufficient response to hospital demand (R40) are all influenced by other risks. As is well known, these risks are classified as operational risks, and risk mitigation strategies are typically used to mitigate them. Of course, depending on the budget available to the supply chain, an acceptable level of shortages or delays can be tolerated, and determining this level of tolerance can be a worthwhile project for supply chain managers.

### 5.2. Managerial and Practical Insights

In many countries, particularly in developing countries, health-care systems face numerous challenges due to a lack of adequate health-management systems and procedures. This is especially true for the blood supply chain and other vital products. It is costly for countries to replace their ageing systems and infrastructure in health systems, so they should plan for unexpected events such as natural disasters and an exponential rise in demand. This demonstrates that, while many risks in the blood supply chain are rare, preparing current health systems is critical. As a result, this research can assist some organisations, such as the Iranian Blood Transfusion Organization, in developing a central system to manage risks associated with the blood supply chain. To help with risk management, the proposed approach can take into account a wide range of realistic factors. Furthermore, this research may benefit the World Health Organization (WHO), which works to promote health, keep the world safe, and assist the vulnerable on a global scale. As previously stated, while many management techniques are now aimed at improving health systems, these approaches are unprepared to deal with risks associated with the blood supply chain. Current risk management techniques emphasise increasing the capacity to respond to challenges, but many risks in blood supply systems cannot be addressed through capacity expansion or the establishment of new infrastructure.

### 5.3. Limitations and Future Directions

The constraints of time and resources affect every research study. Even though each study has its own set of limitations, they can still lead to new discoveries and ideas. The conclusions of this study are the result of interactions between participants and the researcher, as well as the researcher’s observations and interpretations based on some novel approaches. It might be claimed that the qualitative findings of this study should be changed to be generalizable in light of the new situation. Another limitation of this study is related to its boundary. Only those directly participating in the BSC process were chosen. The study did not include all of the critical external stakeholders who are important in improving hospital service delivery. Furthermore, since the purpose of the present study was to examine the relationship among risks and also structure them, the mutual effects of risks were not considered in the SNA approach; otherwise, employing an alternative approach is suggested. The use of MCDM methods after SNA rather than ISM can determine the appropriate risk rankings, so evaluating proper strategies for important risks can be done as well. In this study, there was no discussion of suitable strategies for risk reduction, and further research can determine the appropriate strategies for each of the key risks. Future research can also address BSC disruptions and resiliency strategies.

## Figures and Tables

**Table 1:** Summary of previous studies.

	Author’s Name	Year	Approach	Study Area
Healthcare and Blood	Liu [20]	2009	FMEA and Cluster analysis	Other methods
	Boonyanusith and Jittamai [23]	2019	HOR	
	Lu, Teng, Zhou, Wen and Bi [24]	2013	PDCA and FMEA	
	Dehnavieh, Ebrahimipour, Molavi-Taleghani, Vafaee-Najar, Noori Hekmat and Esmailzdeh [25]	2015	HFMEA	
	Najafpour, Hasoumi, Behzadi, Mohamadi, Jafary and Saeedi [26]	2017	FMEA	
	Cagliano, Grimaldi, Mangano and Rafele [27]	2017	(RBS), (RBM), (FMECA)	
	Mora, Ayala, Bielza, Ataúlfo González and Villegas [30]	2019	HOR	
	Achmadi and Mansur [28]	2018	HOR	
	Jaberidoost, Olfat, Hosseini, Kebriaeezadeh, Abdollahi, Alaeddini and Dinarvand [29]	2015	AHP and SAW	MCDM
Other Areas	Curkovic, Scannell and Wagner [37]	2013	FMEA	Other methods
	Troche-Escobar, et al. [42]	2018	ISM	
	Pujawan and Geraldin [33]	2009	HOR	
	Wu, Blackhurst and Chidambaram [32]	2006	AHP	MCDM
	Gaudenzi and Borghesi [31]	2006	AHP	
	Wang, Chan, Yee and Diaz-Rainey [35]	2012	FAHP	
	Song, Ming and Liu [36]	2018	DEMATEL	
	Fazli, Kiani Mavi and Vosooghidizaji [39]	2015	DEMATEL and ANP	
	Junaid, Xue, Syed, Li and Ziaullah [41]	2020	N-AHP and TOPSIS	
	Moeinzadeh and Hajfathaliha [34]	2009	ANP and VIKOR	
	Nazam, Xu, Tao, Ahmad and Hashim [38]	2015	AHP and TOPSIS	
	Fazli, Kiani Mavi and Vosooghidizaji [39]	2015	DEMATEL and ANP	
	Rostamzadeh, Ghorabaee, Govindan, Esmaeili and Nobar [40]	2018	CRITIC and TOPSIS	

**Table 2:** BSC risks.

Segments	Code	Risks
Reception	R1	An insufficient estimate of the amount needed to collect
	R2	Getting inadequate or incorrect clinical information in the application form
	R3	Unavailability of some blood donation centres
	R4	Decreased blood donor satisfaction
	R5	Software problems
	R6	Differences in the quality of products produced by different blood transfusion centres
	R7	Congestion and rush to donate blood
Medical consultation and examination	R8	Failure of donor screening
	R9	Gathering incorrect information from a donor
Blood donation	R10	Lack of quality and safety during blood donation
	R11	Equipment failure
	R12	Mismatch
	R13	Wastes and losses
	R14	Non-calibrated equipment
	R15	The temperature change of the blood bags
	R16	Late delivery
Other provinces	R17	Decreased blood quality during transportation
	R18	Delay in receiving blood
	R19	Getting poor quality blood
Maintenance	R20	Failure to perform preventive maintenance
Producing production	R21	Temperature changes
	R22	Equipment failure
	R23	Non-calibrated equipment
	R24	Software and system problems
	R25	Sources likely to be compromised following an autoclave explosion
	R26	Expiration of products
Test	R27	Error in confirming the blood group
	R28	Undetectable new viruses
	R29	Incorrect confirmation of the sample
	R30	Unsafe disposal of positive units
	R31	Error in data entry
	R32	Temperature changes
	R33	Non-calibrated equipment
	R34	Equipment failure
Blood products storage	R35	Improper blood inventory level
	R36	Shortage of emergency storage units
	R37	Insecure disposal of expired units
	R38	Blood rotting
	R39	Expiration of blood products
Distribution	R40	An insufficient response to the hospital demand
	R41	Delivering wrong blood bag
	R42	Improper blood supply (life expectancy)
	R43	Improper allocation of blood to different centres (in terms of units)
	R44	Non-standard packaging on delivery
	R45	Delays in shipping
	R46	Equipment failure
	R47	Decreased blood quality during transportation
Warehouse	R48	Product corruption
	R49	Lack of materials and equipment (such as kits and bags)
	R50	Excessive items
Hospitals	R51	A mistake in blood compatibility test
	R52	Delay in the use of allocated blood bags
	R53	Waiting for the blood reserved by doctors
	R54	Insufficient blood inventory level
	R55	Improper disposal of expired units or wastes
	R56	Inappropriate assessment of the amount of blood required before surgery
	R57	Blood rotting
	R58	Temperature changes
	R59	Side effects of blood transfusion
Government	R60	Cumbersome rules (such as customs rules)
	R61	Lack of proper budget allocation
	R62	Economic and political effects of sanctions
	R63	changes in the exchange rate
	R64	Inflation
	R65	Energy rate changes
	R66	Financial crises
Suppliers	R67	Selection of inappropriate suppliers
	R68	Delay in dispatch
	R69	Purchase of inappropriate equipment
	R70	Cut-off relationships with suppliers
	R71	Inappropriate contracts
Education and training administration	R72	Inappropriate public education
Broadcasting organization and cyberspace	R73	Inaccurate and false information and false excitement
Certificate companies	R74	Improper implementation of standards
QC	R75	Error in checking tests
	R76	Inadequate quality control of materials
	R77	Improper quality control of products
	R78	Failure to identify discrepancies in the audit
	R79	Not paying attention to the documentation revision
	R80	Not paying attention to the process of quality assurance system development
	R81	Incorrect conduct of validation studies
(IT)	R82	Unauthorized access to organizational information
	R83	Cyber-attacks and hacking
	R84	Failure to server data recovery
	R85	Lack of data transfer between different systems
Environment	R86	Power outage
	R87	Earthquake
	R88	Fire
	R89	Contagious events
	R90	Severe climate change
	R91	Emerging diseases
Society	R92	Changing culture and lifestyle
	R93	Street chaos
Terrorist groups	R94	Terrorist attacks
	R95	War
Human resources	R96	Safety negligence
	R97	Incompatibility of human resources with the goals of the organization
	R98	Low productivity of the employees
	R99	Strike
	R100	Not paying attention to standards and validations
	R101	Lack of succession
	R102	Not saving the knowledge of human resources

**Table 3:** Importance of the identified risks in terms of the degree of centrality.

Risk	Degree	Risk	Degree	Risk	Degree	Risk	Degree	Risk	Degree	Risk	Degree
R1	37	R18	21	R35	36	R52	12	R69	34	R86	34
R2	19	R19	9	R36	34	R53	33	R70	29	R87	38
R3	8	R20	37	R37	12	R54	33	R71	31	R88	31
R4	27	R21	16	R38	32	R55	10	R72	17	R89	30
R5	39	R22	31	R39	32	R56	20	R73	28	R90	22
R6	30	R23	19	R40	37	R57	26	R74	13	R91	30
R7	39	R24	5	R41	22	R58	14	R75	11	R92	28
R8	29	R25	19	R42	25	R59	32	R76	20	R93	16
R9	31	R26	23	R43	27	R60	25	R77	18	R94	26
R10	25	R27	23	R44	13	R61	29	R78	11	R95	33
R11	38	R28	23	R45	19	R62	36	R79	7	R96	18
R12	29	R29	23	R46	33	R63	36	R80	9	R97	12
R13	39	R30	17	R47	14	R64	17	R81	10	R98	51
R14	13	R31	24	R48	30	R65	10	R82	15	R99	8
R15	26	R32	24	R49	37	R66	30	R83	33	R100	38
R16	37	R33	31	R50	14	R67	37	R84	15	R101	31
R17	16	R34	30	R51	28	R68	30	R85	26	R102	38

**Table 4:** Structural self-interaction matrix.

	R98	R7	R100	R102	R67	R13	R40	R11	R35	R62	R49	R1	R5	R63	R16	R20	R87
R98		X	X	X	V	V	V	V	O	O	V	V	X	O	V	V	A
R7	X		V	A	O	X	O	V	O	O	X	O	X	O	V	O	A
R100	X	A		V	V	V	O	V	V	A	V	V	X	O	V	V	A
R102	X	V	A		V	V	V	V	V	O	V	V	X	O	V	A	A
R67	A	O	A	A		V	V	V	V	A	V	V	V	A	O	V	O
R13	A	X	A	A	A		V	V	V	A	O	O	O	A	V	A	A
R40	A	O	O	A	A	A		A	A	O	A	A	A	O	A	A	A
R11	A	A	A	A	A	A	V		V	A	V	O	0	A	V	A	A
R35	O	O	A	A	A	A	V	A		O	A	A	A	O	A	A	A
R62	O	O	V	O	V	V	O	V	O		V	O	O	V	O	V	O
R49	A	X	A	A	A	O	V	A	V	A		O	O	A	O	A	A
R1	A	O	A	A	A	O	V	O	V	O	O		A	O	O	O	O
R5	X	X	X	X	A	O	V	0	V	O	O	V		O	O	A	O
R63	O	O	O	O	V	V	O	V	O	A	V	O	O		O	V	O
R16	A	A	A	A	O	A	V	A	V	O	O	O	O	O		O	A
R20	A	O	A	V	A	V	V	V	V	A	V	O	V	A	O		A
R87	V	V	V	V	O	V	V	V	V	O	V	O	O	O	V	V	

**Table 5:** Final reachability matrix.

	R98	R7	R100	R102	R67	R13	R40	R11	R35	R62	R49	R1	R5	R63	R16	R20	R87
R98	1	1	1	1	1	1	1	1	1	0	1	1	1	0	1	1	0
R7	1	1	1	1	1	1	1	1	1	0	1	1	1	0	1	1	0
R100	1	1	1	1	1	1	1	1	1	0	1	1	1	0	1	1	0
R102	1	1	1	1	1	1	1	1	1	0	1	1	1	0	1	1	0
R67	1	1	1	1	1	1	1	1	1	0	1	1	1	0	1	1	0
R13	1	1	1	1	1	1	1	1	1	0	1	1	1	0	1	1	0
R40	0	0	0	0	0	0	1	0	0	0	0	0	0	0	0	0	0
R11	1	1	1	1	1	1	1	1	1	0	1	1	1	0	1	1	0
R35	0	0	0	0	0	0	1	0	1	0	0	0	0	0	0	0	0
R62	1	1	1	1	1	1	1	1	1	1	1	1	1	1	1	1	0
R49	1	1	1	1	1	1	1	1	1	0	1	1	1	0	1	1	0
R1	0	0	0	0	0	0	1	0	1	0	0	1	0	0	0	0	0
R5	1	1	1	1	1	1	1	1	1	0	1	1	1	0	1	1	0
R63	1	1	1	1	1	1	1	1	1	0	1	1	1	1	1	1	0
R16	0	0	0	0	0	0	1	0	1	0	0	0	0	0	1	0	0
R20	1	1	1	1	1	1	1	1	1	0	1	1	1	0	1	1	0
R87	1	1	1	1	1	1	1	1	1	0	1	1	1	0	1	1	1

## Data Availability

The data used in the study is available with the authors and can be shared upon reasonable requests.
